# Study CAMBIMED: Effects of changes in medication appearance on safety of antihypertensive and hypolipidemic treatments in chronic patients older than 65 years in primary health care

**DOI:** 10.1186/s12889-014-1342-5

**Published:** 2015-03-04

**Authors:** Jesús Mario Arancon-Monge, Alicia de-Castro-Cuenca, Ángel Serrano-Vázquez, Luz Campos-Díaz, Montserrat Díaz-Eraso, Isabel del Cura-González, Elena Polentinos-Castro, Ricardo Rodríguez-Barrientos, Autores Colaboradores: GRUPO CAMBIMED

**Affiliations:** Centro de Salud Paracuellos de Jarama, Gerencia de Atención Primaria, Servicio Madrileño de Salud, CS Tres Cantos I, Sector Oficios 2, 28760 Madrid, Spain; Centro de Salud Jaime Vera. Servicio Madrileño de Salud, Gerencia de Atención Primaria, Avenida de España S/N, Coslada, 28822, Madrid Spain; Centro de Salud El Puerto, Gerencia de Atención Primaria, Servicio Madrileño de Salud, Calle Océano Pacífico 3, Coslada, 28821, Madrid Spain; Centro de Salud Avenida de Aragón, Gerencia de Atención primaria, Servicio Madrileño de Salud, Calle Alcalá, 425, Madrid, 28027, Madrid Spain; Centro de Salud Fronteras, Gerencia de Atención Primaria, Servicio Madrileño de Salud, Calle Puerto de Navacerrada 4, Torrejón de Ardoz, 28850, Madrid Spain; UDM Atención Familiar y Comunitaria Norte, Unidad de Apoyo a la Investigación, Gerencia de Atención Primaria, Servicio Madrileño de Salud, Calle San Martín de Porres 6, Madrid, 28035, Madrid Spain; Red de Investigacion en Servicios de Salud en enfermedades crónicas (REDISECC), Madrid, Spain; Unidad de Apoyo Técnico, Unidad de Apoyo a la Investigación, Gerencia de Atención Primaria, Servicio Madrileño de Salud, Calle San Martín de Porres 6, Madrid, 28035, Madrid Spain; Unidad de Apoyo a la Investigación. Gerencia de Atención Primaria, Servicio Madrileño de Salud, Calle San Martín de Porres 6, Madrid, 28035 Spain

**Keywords:** Patient safety, Medication adherence, Medication errors, Look-alike, Appearance-equivalent

## Abstract

**Background:**

Different studies have investigated the effects that changes in drug appearance have on the control of chronic diseases and drug safety. The main objective of the proposed study is to evaluate if changes in the appearance of the packaging and presentation of drugs having the same active ingredient are related to a decrease in adherence and an increase in usage errors for chronic treatment using antihypertensive (enalapril and amlodipine) and hypolipidemic agents (simvastatin) in patients ≥65 years old, over a one-year follow-up period.

**Methods/Design:**

We propose a multicentric observational longitudinal cohort study with a one-year follow-up period in 8 primary health care centers (PHCC) in the Community of Madrid. 259 patients who are ≥65 years old, hypertensive and/or dyslipidemic, undergoing treatment with enalapril and/or amlodipine and/or simvastatin, and under formal follow-up of chronic patients in primary health care will be selected by simple random sampling. The main outcome variable will be a final combined variable (adherence and medication usage errors). Other included variables will be: sociodemographic and clinical variables of the patient, degree of disease control, drug taken, number of changes in the appearance of each drug by the pharmacy, and the type and frequency of both avoidable and non-avoidable adverse effects during the follow-up period. A descriptive and a multivariate analysis of the variables will be carried out by means of a logistic regression model, using the final combined variable as the dependent variable (error and/or inadequate usage of the drug), and variables shown to be related to it during the bivariate analysis as the independent variables.

**Discussion:**

For drugs of the same active ingredient, the effect that different package appearances and presentation may have on the safety of patients undergoing chronic treatments is unknown under the new legislative framework. There are various initiatives that promote the iso-appearance of drugs: “If they are the same, make them look the same”. It is to be expected that older, multi-medicated patients with chronic pathologies will be the ones under a greater risk of suffering from this problem.

## Background

In 2010 the Spanish National Health System (NHS) paid for 958 million prescriptions according to data from a report by the Spanish Society of Public Health and Health Administration. Due to this massive exposure of the population to drugs, and to a non-negligible percentage of patients who suffer avoidable adverse effects, the risks associated with drug usage acquire an enormous relevance from the public health perspective [1].

In 2011–2012, legislative changes have been introduced in order to lower the pharmaceutical expenses in the NHS that affect drug prescription. The Spanish law Real Decreto-ley 9/2011, from August 19 [2], requires the medical prescription to be made according to active ingredient, official Spanish denomination, or common international denomination. This way, homogeneous groupings are established for the same active ingredient in order to facilitate the interchangeability of drugs. Later, the Real Decreto-ley 16/2012, from April 20, which addresses urgent measures to ensure the sustainability of the NHS [3], requires dispensing the lowest priced drug out of all the pharmaceutical bio-equivalent presentations, among other measures.

Since the entry of these laws into effect, the doctor writes the prescription according to the active ingredient, with some exceptions (therapeutic needs and lower cost), and the final choice for the commercial brand is determined by the pharmacy based on the lowest-cost criteria. Therefore, the pharmacy can make the necessary changes in order to adjust to the economic criteria.

In this new legal framework, the patient can receive multiple presentations of the same drug when periodically renewing a chronic treatment using the same active ingredient, with different shapes, colors, and sizes of pills, excipients, and packages.

Without doubting the proven clinical efficacy of generic drugs or prescription by active ingredient, this situation generates worries in the medical field about the fact that these different presentations of the same active ingredient may increase the risk of drug usage errors in chronic treatments, since multiple changes of commercial brands can be made at the pharmacy [2], which may increase the risk of drug usage errors by patients. According to data from the APEAS study [4], which employed an observational design to study the safety of patients in primary care during standard clinical practice, examining over 96,000 consultations in the country during 2007, the prevalence of errors is estimated to be around 1.01%, with an emphasis on the fact that in 48.2% of the cases the causes were related to medication.

Various authors and organizations have developed recommendations for alerting of this problem [5,6]. The US Institute for Safe Medication Practices (ISMP) recommends generic drugs keep the same appearance as the original brand, or a standard universal labeling be used for bio-equivalent medications. This Institute has a National Program of Medication Errors Notification in Spain (ISMP-España), where errors of mistaking different drugs with similar packages have been recorded, as happened with Renitec® 5 mg and 20 mg and Zocor® 10 mg and 20 mg because of having similar packaging. In Spain, various scientific societies (Spanish Society of Family and Community Medicine, Spanish Federation of Community Nursery, and Spanish Society of Attending Quality) have signed a manifesto under the motto “If they are the same, make them look the same”, demanding actions so that packages of drugs containing the same active ingredient show a common appearance, which has been denominated “iso-appearance” [7].

Different studies have investigated the effects that changes in the appearance of drugs (package, shape, size, color) have on the control of chronic diseases. Of particular relevance is a systematic review by De Craen *et al*. [8], published in British Medical Journal, including 6 clinical trials that evaluated the effect of the color of pharmaceutical drug presentations on various factors, for example relating the drug color to a stimulating or depressive effect on the nervous system, or to the location of the effect. In addition to the placebo effect that different drug colors produce, color changes can confuse the patient, increasing the risk of errors. On the other hand, the change that happens at the pharmacies when dispensing medication with a different look may also affect the clinical control of the disease. The effects of drug appearance changes are centered on 2 mechanisms: medication errors (mistaking drugs, duplicating doses) and lack of adherence to treatment (either due to or independent of medication errors).

Undoubtedly, the most vulnerable population to these possible mistakes is the older, multi-medicated population. The changes in pharmaceutical presentations may mean an extra difficulty since they often suffer from visual deficiencies, and even cognitive ones, which can increase the risk of mistakes [9]. All this can generate confusion, forgetfulness, or duplication of dosages, which worsens adherence to the treatment, and therefore endangers their safety and even disease control [10]. The Spanish Ministry of Health, Social Services, and Equality itself establishes the investigation of the safety of medical treatments in the >65-year-old population as a strategic research line [11].

Lack of adherence to treatment is a problem of great relevance [12]. In the 2003 Geneva convention [13], the World Health Organization (WHO) already warned that only half of chronic treatments are followed correctly, and that adherence to them is an important modifier of the effectiveness of health systems. Among the causes for the lack of success of chronic treatments, the WHO refers to economical and social factors, the medical team assisting the patient, the health system, the characteristics of the disease, the treatment itself, and factors related to the patient. However, there are no studies that have evaluated the effect that repeated presentation changes may have on treatment adherence. Strategies have been proposed to avoid changes in commercial presentation, some of which imply manufacturing drugs with specific shapes (for example, the famous blue diamond of Viagra©), while others suggest directly advertising the specific look of the pill [14].

Hypertension (HTN) and dyslipidemia are two very prevalent chronic pathologies in our environment. The prevalence of HTN in Spain is estimated to be around 35%, and almost doubles in patients over 65 years old (68%); as for hypercholesterolemia, it is around 50% when considering levels of total high cholesterol (HDL > 200 mg/dL or people under pharmacological treatment), or 44% if considering high levels of low density lipoproteins (LDL > 130 mg/dL or under pharmacological treatment) [15]. The 3 most prescribed drugs for these pathologies (enalapril, amlodipine, simvastatin) have been selected for this study, which have a high impact on the NHS due to the volume of prescriptions and the high number of patients who chronically consume them, according to data provided to the study auhors, by the NHS. The possibility of repeated changes at pharmacies for this group of drugs can be high; in the Spanish pharmaceutical market, there are currently 45 different presentations of enalapril 20 mg, 40 presentations of amlodipine 10 mg, and 47 presentations of simvastatin 20 mg [16].

In primary health care, we find ourselves in a suitable framework to be able to observe under conditions of standard clinical practice the real effect of these legislative changes on patients with chronic diseases. The formal follow-up of these patients in primary health care will allow us to study the possible effects that appearance changes may have on adherence, possible usage errors, and clinical control of the disease.

### Aim

The main aim of this study is to evaluate if changes of drugs with different package and/or presentation appearance for the same active ingredient are related to a decrease in adherence and an increase in usage errors, in patients ≥65 years old, undergoing chronic treatment with antihypertensives (enalapril and/or amlodipine) and hypolipidemic agents (simvastatin), over a one-year follow-up period.

The secondary objectives are: to describe the number of drug changes made at pharmacies, estimate the frequency of avoidable and non-avoidable adverse effects during the follow-up period, describe the tolerance of patients to the dispensed drugs, and study if the number of changes is related to a decrease in the degree of control of blood pressure (BP) and/or LDL cholesterol.

## Methods/design

### Design

Observational longitudinal cohort study with a one-year follow-up period.

### Scope

The study includes patients from 8 primary health care centers (PHCC) from the Community of Madrid: 2 urban ones (PHCC Avenida de Aragón, PHCC Benita de Avila), 3 suburban ones (PHCC Alameda de Osuna, PHCC El Puerto, PHCC Jaime Vera), and 3 rural ones (PHCC Paracuellos de Jarama, PHCC Las Matas, PHCC Fronteras), which attend a total population of approximately 205,000.

### Patients

All patients who are over 65 years old, diagnosed with HTN and/or hypercholesterolemia, and included in a program for following-up chronic patients in PHCCs will be included. The patient must be under treatment with at least one of the following drugs: enalapril, amlodipine, and simvastatin, with a stable dose during the previous 3 months, and must give informed, written consent.

The criteria for exclusion are: having difficulty to properly understand written or spoken Spanish, foreseeing an address change and not being ascribed to the PHCC for the following year, and suffering from severe systemic disease or other limitations that prevent the patient from attending the PHCC for the follow-up visits.

### Sample size

Sample size has been calculated to estimate a proportions difference in the degree of adherence to the treatment of 20% between the exposed group (drug changes at the pharmacy) and the non-exposed group. It is estimated that adherence to treatments in the population undergoing chronic treatment is 50%, and the odds ratio (OR) of suffering an event of adherence decrease is double in the group exposed to drug changes (OR = 2) versus the non-exposed one.

For an alpha error of 0.05 and a beta error of 0.20, 237 patients would be necessary. Taking into account the effect of the doctor on adherence of their patients to treatment, it is considered that the outcome of each patient is not independent from other patients from the same cluster, so it is necessary to adjust the sample size because of the design effect. Considering that the number of included patients per doctor is 15, and assuming an intraclass correlation coefficient of 0.01 [17], the required sample size would be 240. If we estimate a 7% loss of patients to follow-up within a one-year period, the final sample size will be 259 patients.

### Sampling

Random sampling will be performed out of the list of patients who fit the selection criteria (≥65 years old, HTN and/or dyslipidemia, and treated with any of the 3 studied drugs) from each of the 30 participating doctors. Randomization will be performed by an independent researcher using computer software to create the randomization sequence (Excel 2007).

### Variables

The main outcome variable is a final combined variable, defined as “having made some drug usage error and/or an inadequate adherence to treatment regimen”.

Error in drug usage is defined according to the classification proposed by Ruiz-Jarabo *et al*. [18]. The type of error will be recorded (wrong dose, wrong frequency), as well as if there was injury to the patient, in which case the type and severity will also be recorded.

Inadequate adherence will be considered if more than one forgotten dose is detected during the package counting and/or if any of the answers (Yes/No) to the 4 questions of the Morisky-Green test in its validated Spanish version [19] is wrong. In the case of discrepancies between the Morisky test and the counting, the outcome of the counting will prevail. Since one single forgotten dose is a very stringent level of accomplishment, if no adherence differences are observed, different degrees of adherence (80%, 90% and 110%) will be considered.

### Secondary outcome variables

#### Sociodemographic

Age, sex, level of education (illiterate, without studies, primary studies, secondary studies, superior education), and PHCC.

Related to the prescription and active ingredient:

- Prescribed active ingredient: enalapril, amlodipine, or simvastatin.
- Change of drug presentation at the pharmacy (Yes/No).
- Number of changes per drug.
- Suffering an adverse reaction to the drug (Yes/No), interruption of medication (Yes/No), and the reason for such interruption.
- Number of avoidable and non-avoidable adverse effects during the follow-up period.
- Tolerance of the patients to the dispensed drugs according to their own direct statement.

Clinical and analytical:

- Years of development of HTN and/or dyslipidemia. Presence of pathologies that modify the HTN (nephropathologies, sleep apnea, obesity, and malnutrition). Presence of pathologies that modify the lipid profile (hyperthyroidism, hypothyroidism, obesity, and malnutrition). Comorbidity (cardiovascular pathology, neurological pathology).
- Treatments that modify BP and the lipid profile (diuretics, alpha and beta blockers), anti-inflammatories, antidepressants and anxiolytics, thyroxin, hormone replacement therapy. Other concomitant treatments.
- Figures of systolic blood pressure (SBP) and diastolic blood pressure (DBP) in each visit. Difference of SBP and DBP between the first visit and the last recorded data. Percentage of patients with good control at the beginning and at the end of the study.
- Total HDL cholesterol, LDL cholesterol, and triglycerides, both initial and after one year.
- Initial and final weight. Initial height. Difference in body mass index (BMI) between the first and final visits.

### Data collection

**Figure 1:**
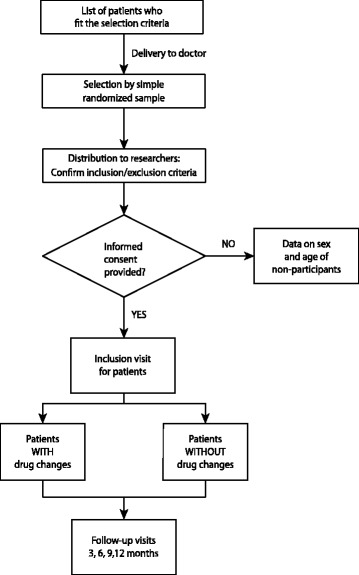
**General scheme of the study.** Randomization and patient follow-up.

Recruitment of patients will be done by their doctor (a study researcher) during a primary care consultation, who will collect the data for the various variables by means of a personal interview and review of the computerized clinical record, after having obtained informed consent, and will record it in an electronic notebook for data collection (Figure 1).

The study entails 5 visits during the trial period, set at 0, 3, 6, 9, and 12 months. Table 1 describes the visits scheme and the variables and measurements to be recorded during each one.

**Table 1:** **Visits and procedures to be performed**

	***Visit*** **1** ***Screening*** **(0 months)**	***Visit 2*** **(3 months)**	***Visit 3*** **(6 months)**	***Visit 4*** **(9 months)**	***Visit 5*** **(12 months)**
Recruitment following the selection criteria	X				
Informed consent	X				
Initial collection clinical information and sociodemographic data	X				
BP control	X	X	X	X	X
Lipid profile control	X		X		X
Counting of drug packages		X	X	X	X
Morisky-Green test	X	X	X	X	X
Data collection for errors and adverse effects		X	X	X	X
Collection of followed-up clinical variables		X	X	X	X

If adverse effects are detected, the notification system for adverse reactions will be followed according to standard clinical practice.

In the case that one patient does not attend a visit, at least 3 attempts to contact them by phone will be performed during different days and hours. Patients lost to follow-up and abandonments will be recorded and the reasons noted.

### Data analysis

Data will be reviewed prior to analysis to guarantee its quality. A descriptive analysis will be performed on the characteristics (age and sex) of patients who refuse to participate in the study and the reasons for it will be described.

A descriptive analysis of each of the variables will be carried out. The mean and typical deviation will be calculated in the case of symmetric distributions, and median and interquartile ranges will be estimated if the distribution is asymmetric. For categorical data, the frequency and percentage distributions will be shown. The difference of proportions will be estimated with its relevant confidence interval of 95%.

The association of each of the independent variables with the dependent variable (adherence decrease and medication errors) will be studied, using Pearson’s Chi-square test or Fisher’s exact test (when the conditions to apply Chi-square are not satisfied) as the statistical tools to compare proportions, and the Student’s T distribution or the Mann–Whitney U non-parametric test to compare the means.

A multivariate test with a logistic regression model will be performed, using the final combined variable (adherence and medication errors) as the dependent variable, and using as independent variables those identified to be associated in the bivariate analysis. All the hypothesis testing will be performed for all analysis’, and a p < 0.05 will be accepted as significant. All the analysis methods will be adjusted considering cluster randomizations [20]. Statistical data analysis will be performed using SPSS 18.0 software.

### Ethical considerations

This study has been approved by the Clinical Research Ethics Board of the Hospital de La Paz (February 5, 2012) in Madrid, and by the Central Research Board of Primary Health Care of the Community of Madrid.

The study will be carried out in accordance with Spanish law, complying with regulation on protection of personal data and patient’s autonomy law. Patients will receive information about the study, both written and oral, and written consent will be obtained from all the participants prior to their inclusion in it.

## Discussion

In our environment, the effects that new legislative measures related to drug prescription may have on the safety of the patient are unknown. For chronic treatment, the interchangeability of drugs with different package appearances and presentations for the same active ingredient may lead to mistakes when choosing the package, or to dose duplications, as well as affecting adherence to treatment by not taking or inadequately taking the medication. These errors may be much more frequent in more vulnerable populations, such as elderly or multi-medicated patients.

Patients will be included and monitored in the study by their own primary care doctors according to standard practice. Including patients from different health professionals implies an increase of variability on the one hand, but on the other hand guarantees a lower number of losses to follow-up since they are chronic patients over 65 years old who periodically attend the PHCC, therefore enabling the analysis of the outcome under conditions of real clinical practice.

It must be highlighted that, in Spain, the majority of the population with the most prevalent chronic pathologies, such as HTN and dyslipidemia, is attended and supervised in primary health care.

In order to decrease the variability among health professionals, training sessions will be held and the protocol for the follow-up of hypertensive patients in primary health care in Madrid will be respected, having been implemented in the data collection notebook. Homologated, calibrated blood pressure meters will be used in order to guarantee the reliability of BP measurements.

We consider that despite the limitations that an observational study may have for answering our question, namely the lack of feasibility of performing a clinical trial as a result of not being able to randomize the intervention (interchangeability of drugs at the pharmacy), it will provide information on the possible effects that the recent legislative changes may have on patient safety.

The management of pharmaceutical services must be focused on both clinical and economic aspects, and must encompass all levels: prescription, dispensing, patients drug intake, and even the manufacturing by following the principle “if they are the same, make them look the same”. The legislation changes based only on cost reduction and forgetful about improvement in medical prescription and pharmaceutical service may worsen the clinical situation and increase the consumption of resources in the long term. The most important is that the patient be the real aim for the outcome of all measures taken to improve their care.

## Abbreviations

BMI: Body mass index
BP: Blood pressure
DBP: Diastolic blood pressure
HDL: High density lipoproteins
HTN: Arterial hypertension
LDL: Low density lipoproteins
NHS: National health system
OR: Odds ratio
PHCC: Primary health care center
SPSS: Statistical package for the social sciences
SBP: Systolic blood pressure
WHO: World Health Organization
